# Gender differences in perceived legitimacy and status perception in leadership role

**DOI:** 10.3389/fpsyg.2023.1088190

**Published:** 2023-05-18

**Authors:** Hyunjin Cha, Yukiko Uchida, Eunsoo Choi

**Affiliations:** ^1^School of Psychology, Korea University, Seoul, Republic of Korea; ^2^Institute for the Future of Human Society, Kyoto University, Kyoto, Japan

**Keywords:** social status, legitimacy, gender gap, leadership, gender inequality, Japan

## Abstract

The present study examined the difference between women and men in perceiving leadership roles. Two experiments, one conducted online and the other in a lab, investigated the subjective experiences of Japanese men and women when they are assigned with different roles (e.g., leader vs. subordinate). Both studies revealed that women perceived their role as less legitimate when they were assigned leader role (vs. subordinate role). In contrast, men did not differ in their perceived legitimacy according to the assigned roles. This discrepancy in legitimacy perception in response to different roles between men and women accounted for a significant variance in women’s lower sense of status when they were a leader (vs. subordinate), but not among men. Our study results illustrate the psychological barrier operating for women in organizations that are embedded in a cultural context in which women leaders are highly underrepresented.

## 1. Introduction

Women leaders are still underrepresented everywhere around of the world (Catalyst, 2020; Gender Equality Bureau Cabinet Office, 2020), even though the proportion of women workers have increased steadily (World Economic Forum, 2022). In particular, Japan’s gender gap in economic participation and opportunity (116th; World Economic Forum, 2022) is especially notable considering its advanced economy (Nakamura and Horimoto, 2021). The driving force of gender inequality in economic activity in Japan is a large gender pay gap, which is primarily caused by a dearth of women in leadership positions (Yamaguchi, 2019). Only 11.2% of managerial positions are women, though women represent more than 69.6% of the workers (Gender Equality Bureau Cabinet Office, 2020).

Despite the growing importance of tackling gender inequality in leadership positions, little attention has been given to the experiences that women leaders go through. In a recent survey conducted in Japan, 50% of working women in their 20s and 30s did not want a managing position (The Star, 2019), suggesting that being in a leadership position for women in such gender unequal society is associated with different meanings and experiences compared to men. However, while much research has been done on what others think “about” women (vs. men) leaders, little research has focused on the experiences of women of leadership *per se* (Karelaia and Guillén, 2014). Existing research that contributed to understanding women leaders’ perspectives are mostly qualitative (Karelaia and Guillén, 2014; Meister et al., 2017; Einarsdottir et al., 2018). In this research using quantitative experimental methods, we aim to compare Japanese men and women’s perception of their own leadership, specifically their sense of status and legitimacy of their role. Japan provides a unique and opportune context in which a wide gender inequality exists in leadership sector and traditional gender roles persists (Kage et al., 2019).

The present study investigated the gender differences in the effects of taking on a leadership role on one’s sense of status and legitimacy. Importantly, we examined whether perceived legitimacy would account for the expected gender differences in the effects of a leadership role on sense of status. Across two studies, we manipulated the leadership role (vs. subordinate) of men and women. We tested whether the formal role affects their sense of status and legitimacy. While Study 1 was conducted online with Japanese adults, Study 2 was conducted in a controlled laboratory setting with Japanese college students to manipulate the leadership more realistically.

## 2. Theoretical background

### 2.1. Psychological challenges for women leaders

Women leaders face unique psychological challenges that men do not (Williams and Tiedens, 2015; Lyness and Grotto, 2018). According to role incongruity theory (Eagly and Karau, 2002), agentic traits of leadership positions are perceived to be incongruent with the traditional traits of women. Women are stereotypically considered more communal than agentic and are thus perceived to lack fit with leadership positions that require agentic traits such as assertiveness and confidence (i.e., “lack of fit model,” Heilman, 1983, 2001). When women do earn a leader position and behave in an agentic way, the lack of fit between gender and leadership stereotypes backfires; they receive negative evaluation for violating stereotyped gender roles (Rudman, 1998; Rudman et al., 2012).

Women in leadership appear to be aware of and responsive to these social evaluations based on gender. Research shows that women leaders feel pressured to manage others’ perceptions about them (Swann et al., 2009; Meister et al., 2014, 2017; Bell et al., 2016) and are more sensitive to others’ evaluations of them than male leaders (Brescoll, 2011). These gender stereotypes have real-world consequences as anticipated discrimination decreased women’s ambitions for leadership (Fisk and Overton, 2019). Women accepted leadership positions less when they expected lower support as a leader (Rink et al., 2012). Moreover, women need higher qualifications than men for promotion (Lyness and Heilman, 2006). In line with this, women do not apply for jobs unless they met all requirements, not because they lack confidence or self-efficacy (Sturm et al., 2014; Taylor et al., 2016), but because they anticipate low levels of acceptance from others (Mohr, 2014). Based on what the role incongruity theory and lack of fit model predict and on extant findings that women in leadership are more sensitive to others’ response than men, we expected that women and men would experience a different sense of status and legitimacy when they are in a leadership position.

### 2.2. Gender differences in sense of status and legitimacy

Social status is defined as the amount of respect received from others (Magee and Galinsky, 2008). Compared to power which leads to focusing on self, status often motivates people to monitor their own status in relation to others and to attend to others’ evaluations (Anderson et al., 2015; Blader et al., 2016). As status characteristics theory (Berger et al., 1977; Ridgeway, 1991) delineates, status is a position embedded in the social structure, and women are assigned with lesser amount of respect than men in our society (Lucas, 2003). Indeed, there are many empirical findings that show people do not respect women in leadership positions as much as they respect men in the same position (Eagly and Karau, 2002; Ragins and Winkel, 2011; Vial et al., 2016). Taking into account that women take up lower status and that individuals are aware of such social reality, we expected that women and men will sense their status differently even when given the same roles.

One possible mechanism may be related to perceived legitimacy. Legitimacy is the belief that “authorities, institutions, and social arrangements are appropriate, proper, and just” (Tyler, 2006, p. 376). In the leadership context, the appropriateness and justness of one’s high position could be defined as legitimacy. Legitimacy perceptions are known to be shaped by the fairness of procedures of status attainment and decision-making by authorities (Tyler, 2000; Tyler and Blader, 2005). However, procedural justice is not the only driving force behind legitimacy; legitimacy is a social process (Johnson et al., 2006), which heavily relies on cultural beliefs about what is natural and ought to be (Major, 1994). For instance, believing that it is natural for men to be leaders and women to be subordinates will legitimize the unequal gender proportion in leadership positions (Pratto et al., 1997). Social dominance theory (Sidanius and Pratto, 1999; Sidanius et al., 2017) proposes that people who are more motivated to accept group-based dominance, that is, people with high social dominance orientation, will endorse legitimizing myths that some groups are more suited for leaders. Extensive research on social dominance theory in organizational context reveals that not only men who enjoy higher status but also women high in social dominance orientation may legitimize gender-based inequality (Tesi et al., 2019, 2020). While it could be counter-intuitive, women with social dominance orientation are willing to uphold unequal gender system and maintain their low status. Beliefs that legitimize the unequal systems are also explained by system justification theory (Jost and Banaji, 1994). Complementary gender stereotypes that present men as agentic and women as communal contribute to justifying gender inequality (Jost and Kay, 2005).

Despite women’s leadership positions, women may feel that they are not qualified enough and do not deserve the position they are given. Women may internalize negative self-perceptions as leaders (Begeny et al., 2021) and become subject to self-fulfilling prophecy (Rosenthal and Jacobson, 1968). Constantly being expected to be in a low position could lead to lower performance as a leader, which will confirm the stereotype that women are not suitable as leaders. Moreover, women’s underrepresentation in leadership positions can visibly signal that women are not suitable for those positions (Ely et al., 2011). It is only natural that women leaders feel inadequate where there are explicit and implicit expectations that women are more suitable for followers but not for leaders (*cf.* imposter phenomenon; Clance and Imes, 1978). Thus, we expected that it will be harder for women to feel legitimacy of their position as a leader.

Furthermore, we explored to what extent and to whom perceived legitimacy and sense of status are interrelated. Attainment and effective use of status greatly depends on legitimacy (Tyler, 2006). Considering that individuals are more likely to “own their role” once they believe they deserve it, the perceived legitimacy of one’s role as a leader would serve as a basis for one’s sense of status in general. In particular, as women face sociocultural barriers of constant feedback that they are not a good fit for leadership roles, they might rely on legitimacy perceptions to sense their status. In other words, women leaders are described as caught in the ‘self-reinforcing cycle of illegitimacy (Vial et al., 2016)’; their low perception of legitimacy is highly likely to be contributing to low status perceptions. Based on the above, the following hypotheses are proposed:

Hypothesis 1: While men would feel a heightened social status when they are in a leader (vs. subordinate) position, women’s sense of status would not be significantly heightened.

Hypothesis 2: While men’s perceived legitimacy will not differ by roles, women’s perceived legitimacy will decrease when they are assigned a leadership (vs. subordinate) role.

Hypothesis 3: For women, legitimacy perceptions will mediate the relationship between assigned role and status perceptions, while for men, the same mediation effect will be insignificant.

## 3. Study 1

### 3.1. Methods

#### 3.1.1. Participants and procedure

Participants were 353 Japanese employees over the age of 19 recruited online through a Japan-based research company called dataSpring.^1^ Three participants who did not report their gender were excluded since we were interested in the role of gender. A total of 350 participants (175 women, 175 men, *M*_age_ = 46.27, *SD*_age_ = 11.08) were included in the final analyses. Mean yearly income level was 3.76 (*SD* = 1.76) on an 8-point scale (1: 0 ~ 2 million yen, 8: over 14 million yen, each scale has a gap of 2 million) which is about 5.52 million yen (3: 4 ~ 6 million yen, 4: 6 ~ 8 million yen). After providing informed consent, participants were randomly assigned to one of two conditions (leader vs. subordinate). They then read the instruction as follows:

*‘Please answer the questions below imagining that you are working as a team member for an average Japanese company. (The questions below do not pertain to your actual job position or affiliation.)’*

This instruction was intended to make participants to imagine the experimentally assigned role as a leader or a subordinate in a very typical Japanese company, rather than referring to their actual role in their workplace when responding. Depending on the condition, participants were asked to imagine that they were either a leader or a subordinate of a team in an ordinary Japanese company. Next, participants then reported their perception of the status and legitimacy of their assigned role.

#### 3.1.2. Measures

##### 3.1.2.1. Perceived status

Perceived status was measured with seven items constructed from existing literature on social status (Fast et al., 2012; Bendersky and Shah, 2013; Hays and Blader, 2017). Sample items were “my social status would be high,” “my team members will respect me.” Participants who were assigned to the leader condition responded to items such as “If I were a leader in my team, my social status would be high.” Participants who were assigned to the subordinate condition responded to items such as “If I were a subordinate in my team, my social status would be high.” Cronbach’s alpha of perceived status was 0.93.

##### 3.1.2.2. Perceived legitimacy

Perceived legitimacy was measured with three items constructed from existing literature on legitimacy (Tyler, 2006). Sample items were “My position would be legitimate’, ‘I would think my position is appropriate.’ Participants who were assigned to the leader condition responded to items such as ‘If I were a leader in my team, my position would be legitimate.’ Participants who were assigned to the subordinate condition responded to items such as ‘If I were a subordinate in my team, my position would be legitimate.’ Cronbach’s alpha of perceived legitimacy was 0.93.

### 3.2. Results

#### 3.2.1. The effect of role and gender on status

First, 2 (gender: women vs. men) × 2 (role: leader vs. subordinate) analysis of variance (ANOVA) was conducted to examine the effects of gender and role assignment on perceived status. There was no significant interaction effect between gender and role, *F*(1, 346) = 0.14, *p* = 0.712, *η*_p_^2^ < 0.001. Since interaction effects were not found, we conducted independent t-tests to determine if there are role differences in status perception in each gender group. For women, there were no differences in status perception according to roles, *M*_leader_ = 3.14, *SD*_leader_ = 0.96; *M*_subordinate_ = 3.10, *SD*_subordinate_ = 0.95; *t*(173) = 0.26, *p* = 0.797, *d* = 0.039. Unexpectedly, neither did men perceive their status differently by their roles, *M*_leader_ = 3.23, *SD*_leader_ = 0.86; *M*_subordinate_ = 3.12, *SD*_subordinate_ = 0.98; *t*(173) = 0.80, *p* = 0.424, *d* = 0.121. Since there were no differences in status perception by position in both gender groups, we could not find support for *Hypothesis 1*.

#### 3.2.2. The effect of role and gender on perceived legitimacy

**Figure 1:**
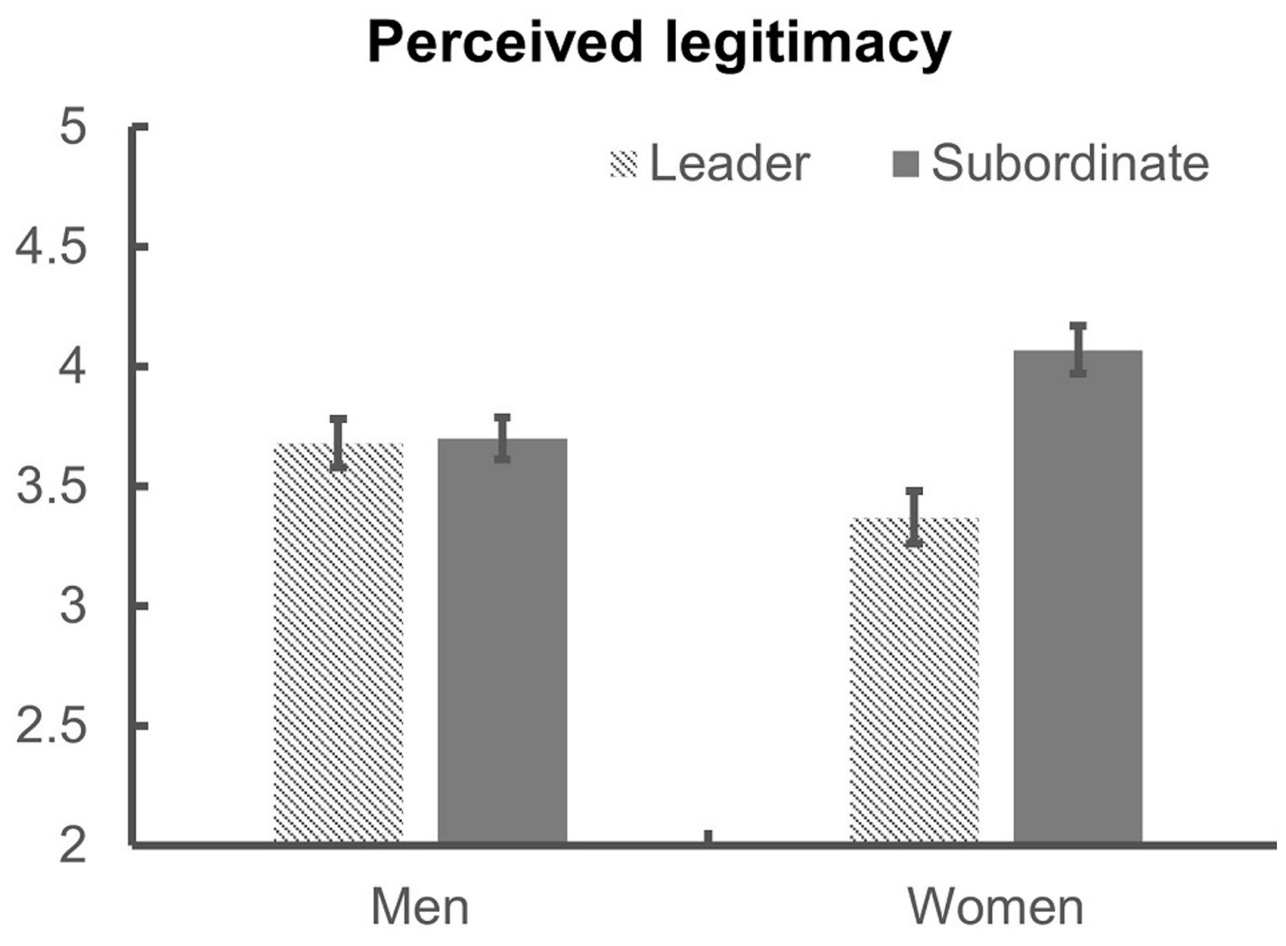
Perceived legitimacy of men and women according to assigned roles in Study 1. Error bars represent standard errors.

Next, 2 (gender: women vs. men) × 2 (role: leader vs. subordinate) analysis of variance (ANOVA) was conducted to examine the effects of gender and role assignment on perceived legitimacy (Figure 1). The interaction effect of gender and role was significant, *F*(1, 346) = 11.19, *p* < 0.001, *η*_p_^2^ = 0.031. Simple main effect analyses showed that, for men, no difference in perceived legitimacy was found between the leader (*M* = 3.68, *SD* = 0.95) and the subordinate condition (*M* = 3.70, *SD* = 0.84), *F*(1, 346) = 0.03, *p* = 0.853, *η*_p_^2^ < 0.001. However, for women, perceived legitimacy was higher in the subordinate (*M* = 4.07, *SD* = 0.92) than in the leader condition (*M* = 3.37, *SD* = 1.05), *F*(1, 346) = 24.16, *p* < 0.001, *η*_p_^2^ = 0.065. This result confirmed *Hypothesis 2* that, unlike men, women would not perceive their leadership role as more legitimate than their subordinate role. In fact, it was the other way around.

#### 3.2.3. The mediating role of legitimacy

Finally, we tested for the mediating role of perceived legitimacy in the effect of role (leader vs. subordinate) on sense of status, and whether this differ by gender. Gender (1: men, 2: women) and role (1: leader, 2: subordinate) were dummy-coded. Initially, the simple mediation analysis using PROCESS Model 4 showed that the mediating effect of perceived legitimacy in the effect of role on sense of status was significant (*b* = 0.20, *SE* = 0.06, 95% CI = [0.092, 0.322]). Next, the full conditional indirect effect model was tested by using PROCESS Model 7. First, the analysis revealed that the effect of role on perceived legitimacy differed by gender. Specifically, the role did not influence perceived legitimacy for men, however, women who were assigned to be a leader (vs. subordinate) reported lower perceived legitimacy. Perceived legitimacy, in turn, predicted sense of status when role, gender, as well as their interaction were controlled for. Overall, there was a significant index for a conditional indirect effect, *b* = 0.38, *SE* = 0.12, 95% CI [0.154, 0.604]. As predicted in *Hypothesis 3*, the perceived legitimacy did not mediate the relationship between the role and sense of status for men (*b* = 0.01, *SE* = 0.08, CI [−0.134, 0.171]). In contrast, perceived legitimacy significantly accounted for the relationship between role and status perception for women (*b* = 0.39, *SE* = 0.09, CI [0.233, 0.571]). The direct effect of role on status perception was significant, *b* = −0.28, *SE* = 0.08, *p* = 0.001.

Overall, the manipulation of role (leader vs. subordinate) did not have any effect on sense of status for both men and women. Considering that Study 1 was conducted online, the manipulation may not have been strong enough to alter sense of status. In Study 2, to create a more realistic setting, we invited a pair of participants to the laboratory and made them think that one of them would be taking on the role of a leader and the other a subordinate.

## 4. Study 2

### 4.1. Methods

#### 4.1.1. Participants

Participants (*N* = 121) were recruited in a university in Japan as part of a larger study. Ten participants who were suspicious of the deception were excluded from the analysis. The final sample size was 111 (46 women, 65 men, *M*_age_ = 20.82, *SD*_age_ = 1.81).

#### 4.1.2. Procedure

A pair of same-gender participants were invited to the lab. Once the participants saw each other, they were immediately guided into two separate rooms so that they have minimal contact with each other. The participants were asked to fill out the background questionnaire asking about leadership experience, academic performance, personality, and mood. After filling out the questionnaire, participants were told that they would be assigned to a position of a leader or a subordinate, based on their response to the questionnaires. In fact, participants’ positions were randomly assigned. Then, participants were told about a joint-task that they and their interaction partner were about to do. The joint-task was on building tangram puzzles, which was found to be effective in manipulating leadership in a previous study (Miyamoto and Wilken, 2010). The leader of the dyad was supposed to provide a direction to build tangram puzzle which the subordinate has to follow. To bolster the manipulation of the role, participants wrote several sentences about how they will participate in the joint-task with their partner. The joint-task was bogus and was never conducted in reality. Participants were debriefed at the end of the study.

#### 4.1.3. Measures

Sense of status was measured with two items, “At this moment in time, I feel high in social rank.” and “At this moment in time, I feel high in social status.” The responses were measured on a 7-point Likert scale (1 = disagree strongly, 7 = agree strongly) (*r* = 0.84, *p* < 0.001). Perceived legitimacy was measured with the degree to which a participant agrees with the sentence, “At this moment in time, I feel that my social rank is legitimate.” on a 7-point Likert scale (1 = disagree strongly, 7 = agree strongly).

### 4.2. Results

#### 4.2.1. The effect of role and gender on status

**Figure 2:**
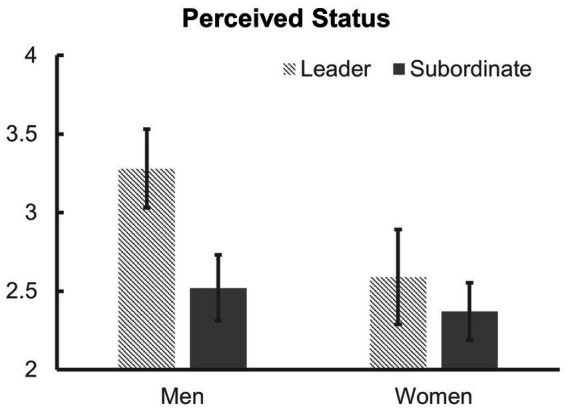
Perceived status of men and women according to assigned roles in Study 2. Error bars represent standard errors.

First, a 2 (gender: women vs. men) × 2 (role: leader vs. subordinate) analysis of variance (ANOVA) was conducted to examine the effects of role assignment on perceived status (Figure 2). There was no significant interaction between gender and role, *F*(1, 107) = 1.22, *p* = 0.271, *η*_p_^2^ = 0.011. Since we did not find an interaction, we conducted independent t-tests to decide if there are role differences in status in each gender groups. Women did not show role differences in sense of status, *M*_leader_ = 2.59, *SD*_leader_ = 1.45; *M*_subordinate_ = 2.37, *SD*_subordinate_ = 0.88; *t*(44) = 0.61, *p* = 0.542, *d* = 0.181. However, as expected, men leaders (*M* = 3.28, *SD* = 1.46) perceived their status higher than men subordinates (*M* = 2.52, *SD* = 1.17), *t*(63) = 2.31, *p* = 0.024, *d* = 0.576. Therefore, *Hypothesis 1* was supported.

#### 4.2.2. The effect of role and gender on perceived legitimacy

**Figure 3:**
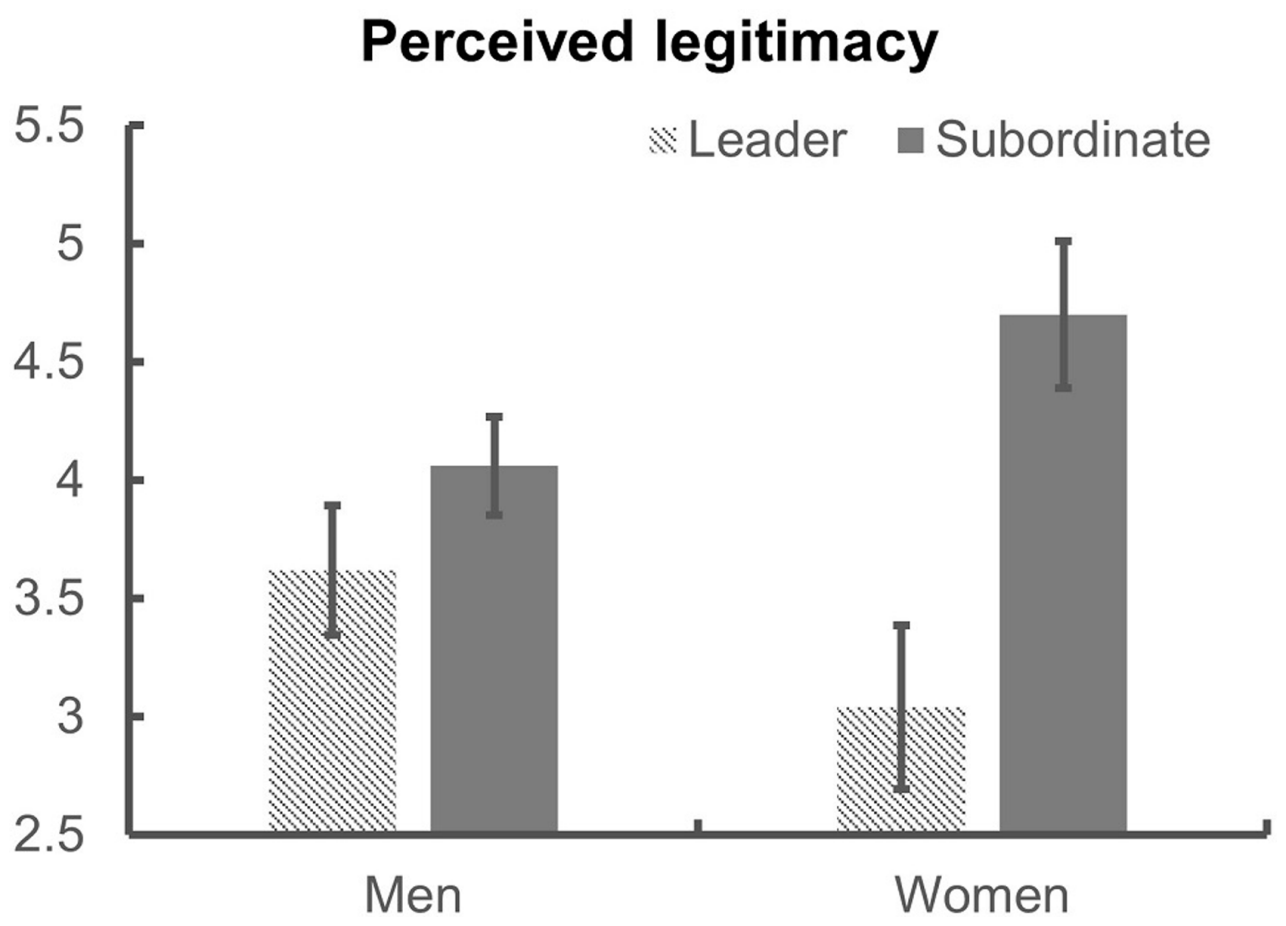
Perceived legitimacy of men and women according to assigned roles in Study 2. Error bars represent standard errors.

Next, we examined whether there are gender differences in perceived legitimacy depending on the assigned roles (Figure 3). A 2 (gender: women vs. men) × 2 (role: leader vs. subordinate) ANOVA revealed that there was a significant interaction effect between gender and role, *F*(1, 107) = 4.48, *p* = 0.037, *η*_p_^2^ = 0.04. A simple main effect analysis was conducted to better understand this interaction. As predicted in *Hypothesis 2*, the difference in the perceived legitimacy was driven by women. Women leaders (*M* = 3.04, *SD* = 1.66) perceived lower legitimacy than women subordinates (*M* = 4.70, *SD* = 1.49), *F*(1, 107) = 14.37, *p* < 0.001, *η*_p_^2^ = 0.118. In contrast, men did not differ in their perceived legitimacy depending on the role assigned, *M*_leader_ = 3.62, *SD*_leader_ = 1.60; *M*_subordinate_ = 4.06, *SD*_subordinate_ = 1.15; *F*(1, 107) = 1.48, *p* = 0.23, *η*_p_^2^ = 0.014.

#### 4.2.3. The mediating role of legitimacy

**Figure 4:**
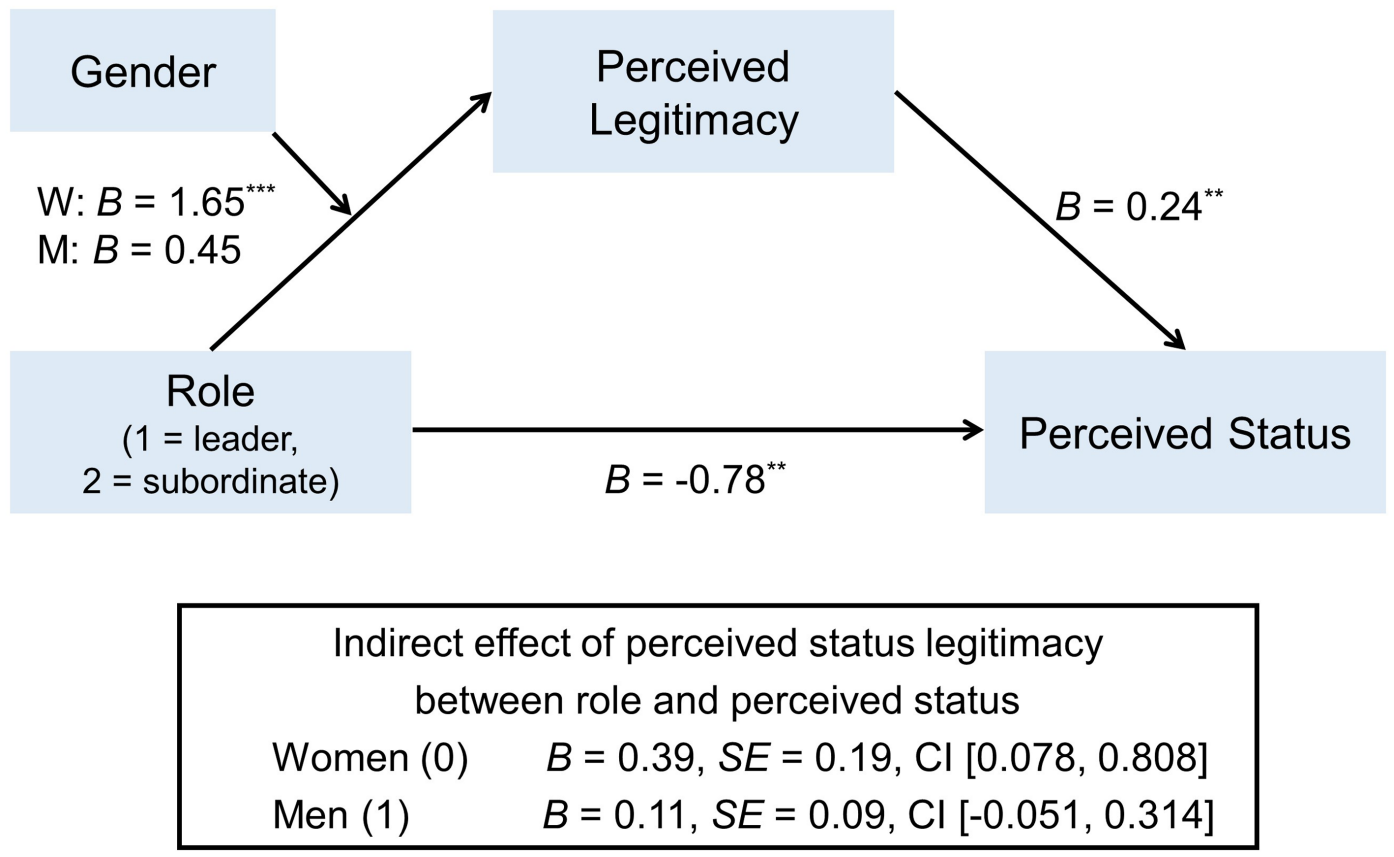
Conditional indirect effect of perceived legitimacy in the relationship between role and sense of status for men and women in Study 2. Role (leader vs. subordinate), gender (women vs. men), ^*^p < 0.05, ^**^p < 0.01, ^***^p < 0.001.

Finally, as in Study 1, we tested for the conditional indirect effect of perceived legitimacy in the relationship between role and sense of status for men and women (Figure 4). Gender (1: men, 2: women) and role (1: leader, 2: subordinate) were dummy-coded. First, the simple mediation analysis using PROCESS Model 4 showed that the mediating effect of perceived legitimacy in the relationship between role and sense of status was significant, *b* = 0.23, *SE* = 0.10, 95% CI = [0.045, 0.473]. Next, the full conditional indirect effect model was tested by using PROCESS Model 7. The analysis revealed that the effect of role on perceived legitimacy differed by gender. Specifically, the role did not influence perceived legitimacy for men; however, women who were assigned to be a leader (vs. subordinate) reported lower perceived legitimacy. Perceived legitimacy, in turn, predicted sense of status when role, gender, as well as their interaction were controlled for. Overall, there was a significant index for a conditional indirect effect, *b* = 0.29, *SE* = 0.19, 95% CI [0.003, 0.713]. As predicted in *Hypothesis 3*, the perceived legitimacy did not mediate the relationship between the role and sense of status for men (*b* = 0.11, *SE* = 0.09, 95% CI [−0.051, 0.314]). In contrast, perceived legitimacy significantly accounted for the relationship between role status perception for women (*b* = 0.39, *SE* = 0.19, 95% CI [0.078, 0.808]). The direct effect of role on status perception was significant, *b* = −0.78, *SE* = 0.25, *p* = 0.002.

## 5. Discussion

In Study 1, role manipulation did not affect the sense of status for either men or women, which may be because the study was conducted online. However, in Study 2, which was conducted in a more realistic setting, only men and not women reported a higher sense of status when they were assigned the role of leader (vs. subordinate). This gender difference was explained by the lower perceived legitimacy of women assigned as a leader than those assigned as a subordinate. In sum, across two studies, women perceived that their role was less legitimate when they were a leader (vs. subordinate), which, in turn, predicted a lower sense of status. However, men did not differ in their perceived legitimacy regardless of their role, and their legitimacy perceptions did not mediate the relationship between role and status. Although speculative, men would not need to reflect on their legitimacy of a role, unlike women who self-monitor, given the male privilege (Case et al., 2012). Legitimacy could be a concept that is accepted by default and only questioned when there are lingering problems like one’s identity being non-prototypical to the position.

These findings are consistent with the prior literature on gender role theory (Eagly, 1987) in organizational settings (Eagly and Steffen, 1984; Eagly and Johnson, 1990). For men, there is congruency between their role and sense of status, whereas the two are disconnected for women. Of note, women thought their role was more legitimate when assigned to be a subordinate than a leader. Interestingly, this was the case even when participants were provided with reasons for their assigned roles (Study 2), albeit bogus. As low legitimacy perceptions of women leaders were an important mediator between roles and status perceptions in the present study, and might lead to precarious and ineffective leadership (Vial et al., 2016), it is important to understand why women leaders have a hard time embracing the legitimacy of their roles.

Social dominance orientation (Tesi et al., 2019) and system justification (Kray et al., 2017) among women could explain the lack of perceived legitimacy of their leadership position. In a society where gender inequality is severe such as Japan, structural changes toward gender equality may be deemed unlikely (Kay and Friesen, 2011). Also, women may deny their legitimacy as leaders as a way to avoid a possible discrimination (Fisk and Overton, 2019). In other words, legitimizing the unfair gender relationships could be an attempt for adjustment and a survival strategy for women (van der Toorn et al., 2015). Alternatively, women may feel more comfortable exerting influence based on other than legitimate power, such as reference power (French et al., 1959; Carli, 1999; Raven, 2008).

Meanwhile, some researchers argue that an organizational setting is a “strong” situation in which roles define clear expectations of behaviors, and thus, the impact of gender norms may be reduced (Deaux and Major, 1987). However, it is important to remember that the present study is conducted in a Japanese context where the proportion of women in managerial and leadership roles is still very small. As mentioned, many young Japanese women on the managerial track express that they do not wish to be a manager. Given that women’s leadership experiences differ in terms of race and nationality (Windsor et al., 2020; Wong et al., 2022), it would be extremely beneficial to explicitly take into consideration the role of cultural factors (e.g., hierarchal and collectivistic culture, gender and inequality) in understanding the unique challenges women leaders face in a social context where even organizational setting is subject to cultural norms regarding gender.

The present study suggests that the strategies that increase legitimacy perceptions of women in higher positions would be critical as low legitimacy perceptions could undermine leadership aspirations and performance (Vial et al., 2016). The strategies that shift gender stereotypes would be especially beneficial. For instance, hiring and promoting more women to increase the representation of women leaders (Lyness and Grotto, 2018), exposing women to more same-gender leadership role models (Dasgupta and Asgari, 2004; Olsson and Martiny, 2018) and supervisors (Fritz and van Knippenberg, 2020), and more same-gender networking opportunities (Yang et al., 2019; Manongsong and Ghosh, 2021; Yeoward and Nauta, 2021) are such strategies that have been found to be effective in reducing gender stereotypes. In addition, promoting the belief that there is no demographic prototype for a leader and that there are leaders with diverse identities is found to be effective for buffering gender bias (Ely et al., 2011; Hoyt and Burnette, 2013; Hoyt and Murphy, 2016), and also for reducing system justification regarding gender inequality (Kray et al., 2017). Lastly, feminism is found to increase leadership aspiration for highly identified women (Leicht et al., 2017). Since feminism urges women to acknowledge and challenge gender inequality, feminism will be effective in challenging illegitimacy perceptions rooted in unequal structure.

The present research is limited in that both Study 1 and Study 2 were conducted in a hypothetical situation. Much research remains to be done with a more ecologically valid methodology, such as longitudinal studies of employees in an organizational setting. However, our study provides insights into how the structural barrier for women leaders may translate into subjective experience. Gender differences in status and legitimacy perceptions found in our study signal the “lack of fit (Heilman, 1983)” that women leaders are likely to be experiencing in organizations. Future research would greatly benefit from identifying specific conditions under which women of leadership do or do not interpret their position as well-founded.

## Data availability statement

The raw data supporting the conclusions of this article will be made available by the authors, without undue reservation.

## Ethics statement

The studies involving human participants were reviewed and approved by KUIRB-2020-0316-01. The patients/participants provided their written informed consent to participate in this study.

## Author contributions

HC: data curation, investigation, formal analysis, and writing – original draft. EC: conceptualization, writing – original draft, and review and editing. YU: data curation, investigation and writing – review and editing. All authors contributed to the article and approved the submitted version.

## Funding

This research was funded by Japanese Society for the Promotion of Science, No. PE14053.

## Conflict of interest

The authors declare that the research was conducted in the absence of any commercial or financial relationships that could be construed as a potential conflict of interest.

## Publisher’s note

All claims expressed in this article are solely those of the authors and do not necessarily represent those of their affiliated organizations, or those of the publisher, the editors and the reviewers. Any product that may be evaluated in this article, or claim that may be made by its manufacturer, is not guaranteed or endorsed by the publisher.

## Footnotes

^1^ https://www.d8aspring.com
